# Supplementary material for: LASP1, CERS6, and Actin Form a Ternary Complex That Promotes Cancer Cell Migration
Source: Cancers (Basel). 2023 May 16;15(10):2781. doi: 10.3390/cancers15102781 (PMC10216351; doi:10.3390/cancers15102781)
Supplement: Supplementary file 1 [file cancers-15-02781-s001.zip › Supplementary Methods.pdf]

Supplementary Methods

Identification of CERS6 binding proteins

SDS-PAGE

Eluted samples were applied to 1-mm thick 15-well plates with 12% SDS-PAGE gel. Samples were separated by about 1 cm, until the entire sample was covered with acrylamide gel.

Cutting

Each lane of gel was cut using the Coomassie brilliant blue (CBB) stain in the sample buffer as a guide. Following electrophoresis, the gel was fixed for five minutes, then each was immediately cut with a clean scalpel.

After cutting

Excised intact gel pieces were transferred into 1.5-mL low-peptide-binding microtubes and fixed for 1 hour with 5% (v/v) acetic acid in 1:1 (v/v) water:methanol, then each rectangular gel piece was transferred to a new well in a 96-well filter plate using tweezers. Using a Biotage® PRESSURE+96 (Biotage, Uppsala, Sweden) positive pressure manifold, the gels were washed with water and dehydrated, then reduced and alkylated.

Digestion

Before digestion, each dried gel was transferred to a new low-peptide-binding microtube using tweezers, then a sufficient amount of protease solution was added to cover half of the dry gel (75 μL). The sample was placed on ice at a 45-degree tilt to facilitate immersion of the gel in the solution, with vortex application and spinning every

30 minutes. After 30-60 minutes, a sufficient amount of 50-mM ammonium bicarbonate (e.g., 225 μL) was added after ensuring the gel was transparent. Samples were incubated overnight at 37°C in a thermomixer with shaking at 300 rpm. The protease solution consisted of Lys-C (150 ng) and trypsin (150 ng) dissolved in 50 mM of ammonium bicarbonate at pH 8.0.

Extract digested peptides and desalting

Following overnight digestion, each sample was acidified with 9 μL of 20% (v/v) trifluoroacetic acid (TFA). A MonoSpin® C18 column (GL Sciences, Tokyo, Japan) was pre-activated using neat acetonitrile (ACN) and equilibrated with water, then the acidified peptides extract was directly loaded into the equilibrated column. Water (250 μL) with 0.1% TFA (v/v) was added to the remaining gel and the sample was incubated at 37°C for 10 minutes with shaking (450 rpm). This second extract was added to the first extract on the column and centrifugation was performed at 2300 xg for 2 minutes. The column was then placed in a clean low-peptide-binding microtube, and the extracts adsorbed on the column were eluted with 200 μL of 50% ACN and 0.1% (v/v) TFA by centrifugation at 2300 xg for 2 minutes.

LC-MS/MS analysis

Dried peptides were resuspended in 20 μL of 2% ACN and 98% water with 0.1% trifluoroacetic acid, then 7.25 μL of each sample was subjected to analysis with a Q Exactive LC coupled to a mass spectrometer using an Ultimate 3000 nanoLC-MS/MS system (Thermo Fisher Scientific). After injection, the peptides were trapped on a 5 × 0.3-mm ID trap column packed with 5 μm of C18 resin and separated at a flow rate of 500 nL/min using a 5-40% buffer B gradient for 100 minutes in a NANO-HPLC capillary column C18 (0.1 × 125 mm, Nikkyo Technos). The composition of LC buffer A was 0.5%

(v/v) acetic acid in water, while that of LC buffer B was 80% (v/v) ACN and 0.5% (v/v) acetic acid in water. Survey full scan MS spectra were collected from 350-1800 m/z in the Orbitrap with a resolution of 70,000 and an AGC target of 3E6. For the MS/MS experiment, the 10 most intense multiplied charged precursors (z ≥2) were accumulated to a 1E5 target value and fragmented in the collision cell by higher-energy collisional dissociation (HCD). The precursor isolation width was 2.0 m/z and HCD normalized collision energy was 27%. The maximum injection time was set to 60 milliseconds and dynamic exclusion was set to 10 seconds.

Data analysis

MS and MS/MS data results were analyzed using label-free quantification [39], and compared with those in a database using Proteome Discoverer 2.4.1.15 (Thermo Fisher Scientific), with the following parameters. Database: UniProt (constrained to *Homo sapiens*) and cRAP for contaminants (http://www.thegpm.org/crap/); enzyme, trypsin; maximum missed cleavage sites, 2; minimum and maximum peptide length, 6 and 144, respectively; variable modifications, oxidation (M); static modification, carbamidomethyl (C); precursor mass tolerance, ± 10 ppm; fragment mass tolerance, ± 0.02 Da; precursor abundance basis, intensity. For label-free quantification, retention time alignment was performed across the samples and peptide abundance was estimated with an MS1 peak intensity-based quantification function using proteome Discoverer 2.4. Based on values for abundance ratio (anti-CERS6 antibody-bound samples vs. those from anti-IgG antibody), co-precipitated protein candidates were sorted (**Table S2**). All experiments were performed twice.

Supplementary References

39. Zhu, W.; Smith, J.W.; Huang, C.M. Mass spectrometry-based label-free quantitative proteomics. *J Biomed Biotechnol* **2010**, *2010*, 840518, doi:10.1155/2010/840518.
