# Supplementary material for: LASP1, CERS6, and Actin Form a Ternary Complex That Promotes Cancer Cell Migration
Source: Cancers (Basel). 2023 May 16;15(10):2781. doi: 10.3390/cancers15102781 (PMC10216351; doi:10.3390/cancers15102781)
Supplement: Supplementary file 1 [file cancers-15-02781-s001.zip › Supplementary Tables and Figures.pdf]

Table S1 Oligonucleotides used in this study.

Experiment	Sequence
**siRNA (forward sequence only)**	
siCTRL	SIC002, MISSION® siRNA Universal Negative Control #2 (Sigma-Aldrich)™
siCERS6-1	r(AAGGUCUUCACUGCAAUUACA) dTdT
siCERS6-2	SASI_Hs01_00204321 (Sigma-Aldrich)™
siCERS6-3	SASI_Hs01_00204322 (Sigma-Aldrich)™
siLASP1-1	SASI_Hs01_00139094 (Sigma-Aldrich)™
siLASP1-2	SASI_Hs01_00139095 (Sigma-Aldrich)™
**CERS6-HA pcDNA3**	
pcDNA *Hin*DIII F5	CGTCGAAGCTTGCCACCATGGCAGGGATCTTAGCC
pcDNA-*Bam*HI HA R5	CGTACGGATCCTCAAGCATAATCTGGAACATCATATGGATAATCATCCATGGAGCAGG
**LASP1-truncated mutants**	
LASP1 F1	GGCGGTCGACGAACCCCAACTGCGCCCG
LASP1 R1	TAGAGCGGCCGCTGCGGGTTCAGATGGCCTCC
LASP1 F2	CACCAAGCTTATGAACCCCAAC
LASP1 R2	CAGTGCGGCCGCTTACTTATCGTCGTCATCCTTGTAATCA<br>GAGCCTCCACCCCCGGATCCCTGCTTGGGGTAGTGTGCG
LASP1 R3	CAGTGGATCCACCTTTGCCCTTGTTC
LASP1 R4	CAGTGGATCCAGGGCCCATGCGGCTC
LASP1 R5	CAGTGGATCCTGGGGCGCTGCGCTGTATGG
LASP1 R6	CAGTGGATCCGATGGCCTCCACGTAGTTG
LASP1 F3	ACCAAGCTTATGTCCTTCACCATGGTGGCG
**Sequencing**	
LASP1 seq F4	CACAGGTGTCCACTCCCAGG
CERS6 seq F1	CCGTGCGCCATAGCCCTC
CERS6 seq F2	GGCCCGAGTAGGAACGCTGG
CERS6 seq F3	GCTGATGCTCTTCTGGAGGC
CERS6 seq F4	GCTTCTGGTCTTACTTGATTG
CERS6 seq R2	TTAATCATCCATGGAGCAGGAG
CERS6 seq R3	GCTAGACTCAATATCACTTC
**qPCR**	
LASP1 qF1	GCAACAGAGTGAGCTCCAGAG
LASP1 qR1	TGAAACCTTTGCCCTTGTTC
CERS6 qF1	CCTCTATCTCGCTTTTCCCCT
CERS6 qR1	CGGAGCAATTTGTGGTCCATT
18SF1	AATCAGGGTTCGATTCCGGA
18SR1	CCAAGATCCAACTACGAGCT

Table S2 **Top 10 proteins with potential interactions with CERS6 and IgG in A549 cells shown by immunoprecipitation, liquid chromatograph, and tandem mass spectrometry (IP and LC-MS/MS) findings.**

Gene name	Synonyms	Description	Abundance ratio	
			Exp 1	Exp 2
LASP1	MLN50	LIM and SH3 domain protein 1	200	200
CERS5	LASS5	Ceramide synthase 5	200	75.9
ATP5H	ATPQ	ATP synthase subunit d, mitochondrial	200	8.85
CERS6	LASS6	Ceramide synthase 6	104	124
IGHV6-1	IGHV61	Immunoglobulin heavy variable 6-1	19.2	24.0
ATP5O	ATPO	ATP synthase subunit O, mitochondrial	15.9	25.0
ATP5F1B	ATP5B, ATPMB, ATPSB	ATP synthase subunit beta, mitochondrial	11.5	8.90
ATP5F1A	ATP5A, ATP5A1, ATPM, ATP5AL2	ATP synthase subunit alpha, mitochondrial	11.0	11.0
ATP5F1	ATP5PB	ATP synthase F(0) complex subunit B1, mitochondrial	8.35	200
SURF4	SURF-4	Surfeit locus protein 4	8.09	200

IP was performed using a Dynabeads™ Co-Immunoprecipitation Kit (Invitrogen 14321D), according to the manufacturer’s protocol. Protein complexes were eluted and subjected to sodium dodecyl sulphate-polyacrylamide gel electrophoresis (SDS-PAGE). After in-gel digestion was performed [16], peptides were analyzed by liquid chromatograph-tandem mass spectrometry (Supplementary Methods). Based on the values for abundance ratio (anti-CERS6 antibody bound samples vs. those from anti-IgG antibody), co-precipitated protein candidates were sorted. Each experiment was performed twice.

Figure S1 RAC1-positive lamellipodia were stained by phalloidin.

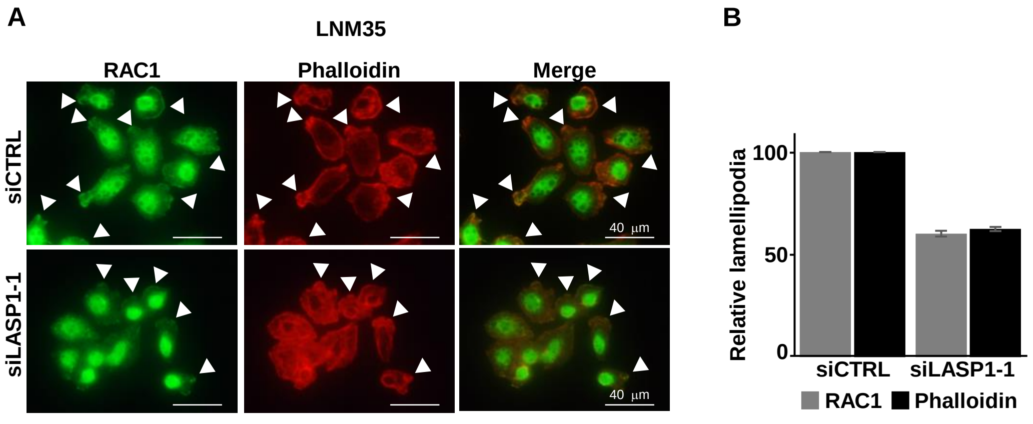

(**A**) Immunocytochemistry analysis using anti-RAC1 antibody (dilution 1:400) and phalloidin (dilution 1:20,000). Scale bar = 40 μm. Arrowhead: lamellipodia. (**B**) RAC1-positive (gray) and phalloidin-positive (black) cells were quantitated. Values relative to those for siCTRL are shown.

Figure S2 LASP1-Flag mutant lacking the N-terminal LIM domain.

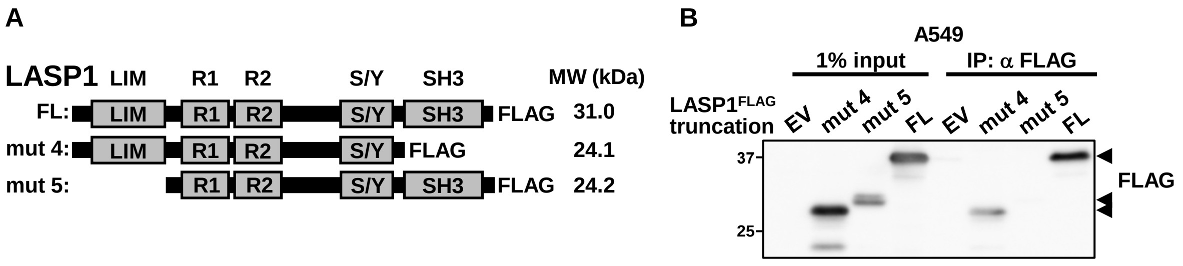

(**A**) Schematic illustration of LASP1-FLAG. Shown are full-length (FL) and two truncated mutants (mut 4, 5). Predicted molecular weights are indicated. (**B**) IP-WB with the anti-FLAG antibody to precipitate LASP1-FLAG. EV, empty pCMV vector (n=2).

Figure S3 Migration and lamellipodia formation efficiencies in A549.

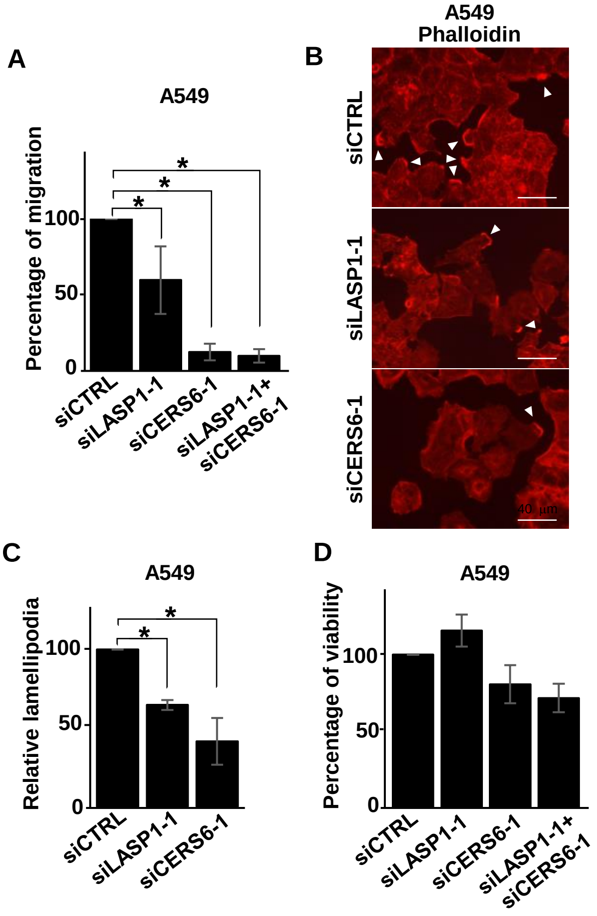

(**A**) Cell migration of A549 cells treated with indicated siRNA(s) was performed. At least 200 cells were counted, with values relative to those for siCTRL shown. *$p<0.05$. (**B**) A549 cells were knocked down as indicated, then fixed and stained with phalloidin. Scale bar = 40 μm. Arrowhead: lamellipodia. (**C**) Quantitative results are shown as values relative to siCTRL. At least 200 cells were counted, with the results are presented as the mean ± SD. *$p<0.05$. (**D**) For the cell viability assay, siRNA-treated cells were seeded at a density of 1 x 10^5 cell/mL on a 96-well plate and cultured under induction conditions. Viable cells were determined using a Cell Counting Kit-8 (Dojindo, 341-07761). All experiments were performed three times.

Figure S4 Protein expression levels of LASP1 and CERS6 in LNM35.

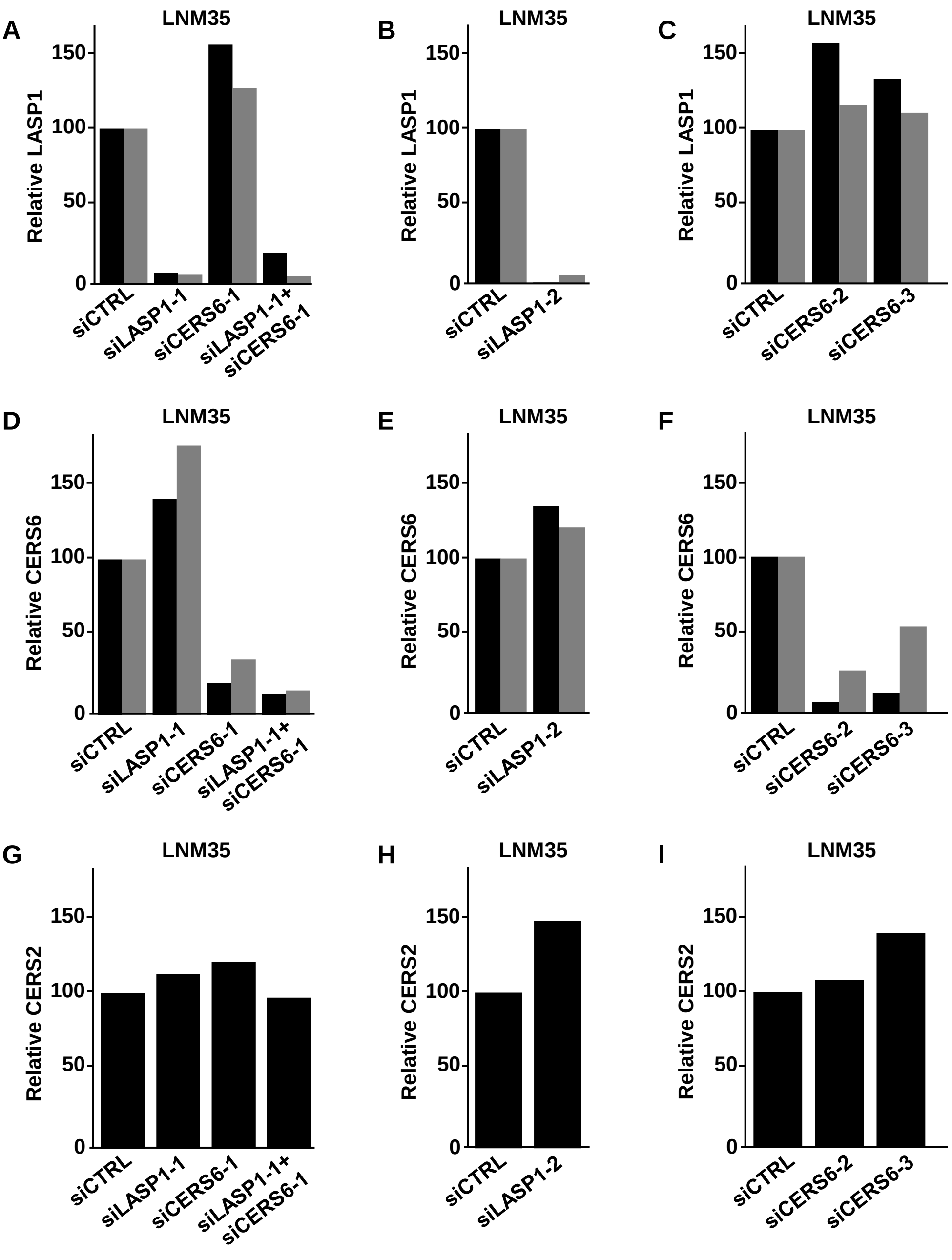

Western blotting analysis was performed using LNM35 cells under the indicated knock-down conditions. LASP1 (**A-C**), CERS6 (**D-F**), and CERS2 (**G-I**) were quantified as compared to siCTRL. Relative values to histone H3 are shown. Black, experiment 1. Gray, experiment 2.

Figure S5 mRNA expression levels of LASP1 and CERS6 in LNM35.

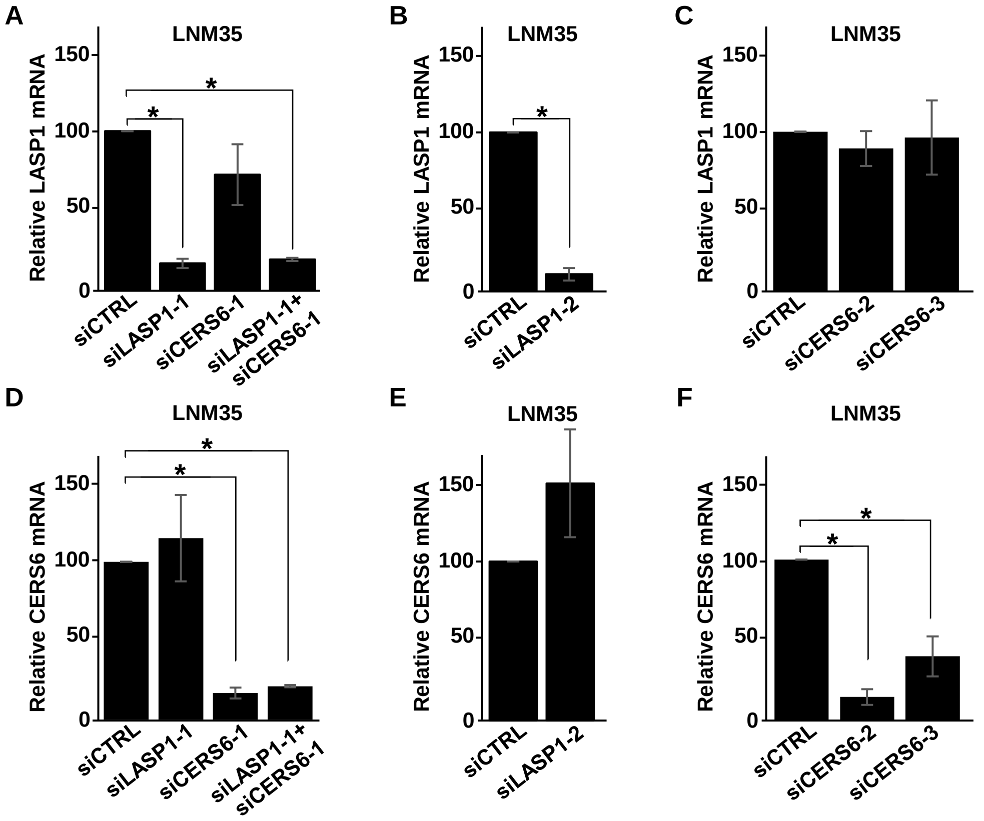

LNM35 cells were knocked down under the indicated conditions. LASP1 (**A-C**) and CERS6 (**D-F**) mRNA levels were determined using quantitative RT-PCR analysis, and plotted values relative to siCTRL. *p<0.05, at least three independent experiments.

Figure S6 Ceramide amounts in A549.

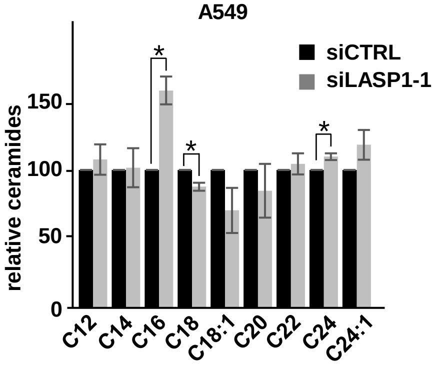

Using LC-MS/MS, ceramide amounts were determined in siCTRL-treated (black) and siLASP1-1-treated (gray) A549 cells (n=3). Quantitative results are shown as values relative to siCTRL. *p<0.05.

Figure S7 Protein expression levels of LASP1 and CERS6 in A549.

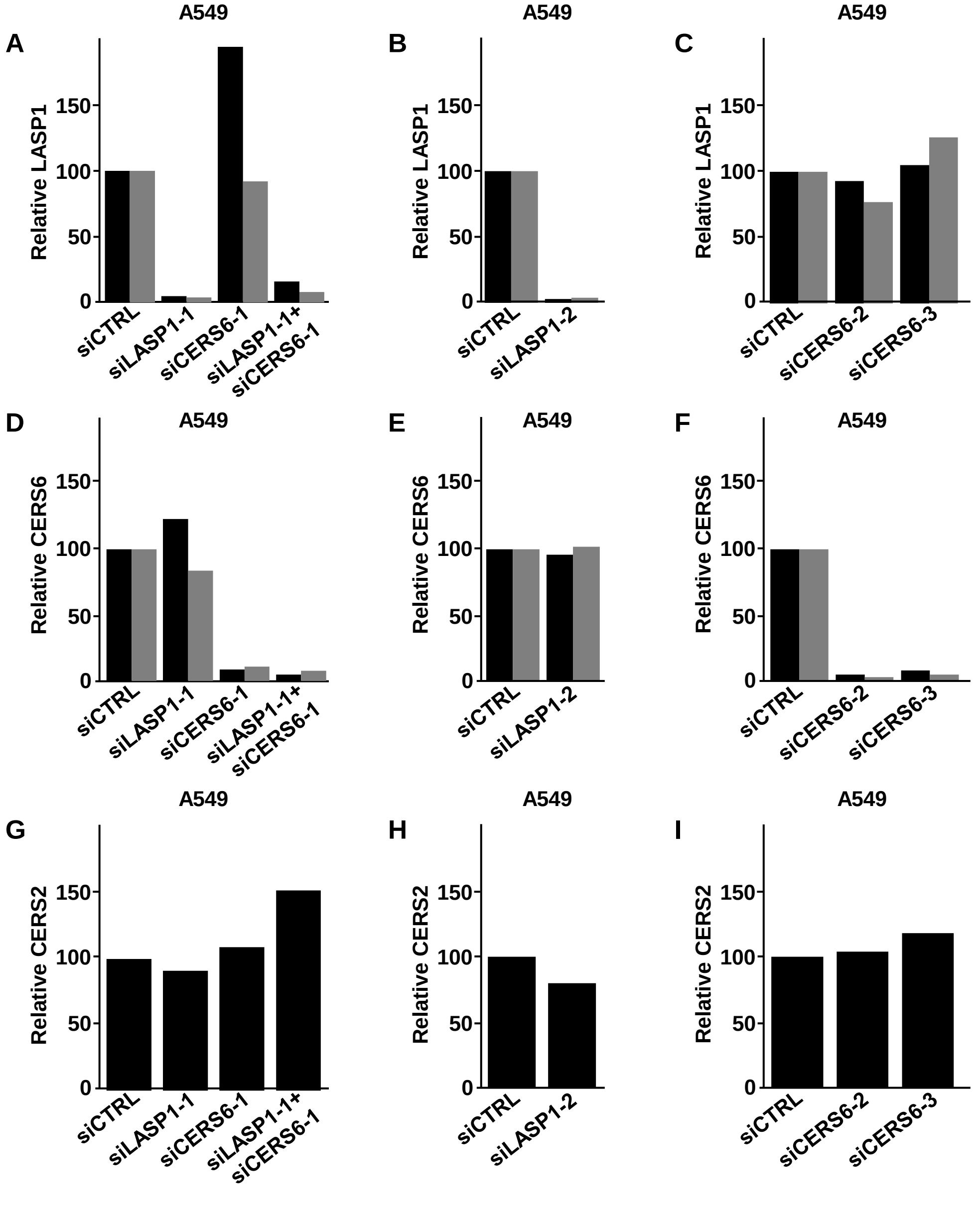

Western blotting analysis was performed using A549 cells under the indicated knock-down conditions. LASP1 (**A-C**), CERS6 (**D-F**), and CERS2 (**G-I**) were quantified as compared to siCTRL. Relative values to histone H3 are shown. Black, experiment 1. Gray, experiment 2.

Figure S8 mRNA expression levels of LASP1 and CERS6 in A549.

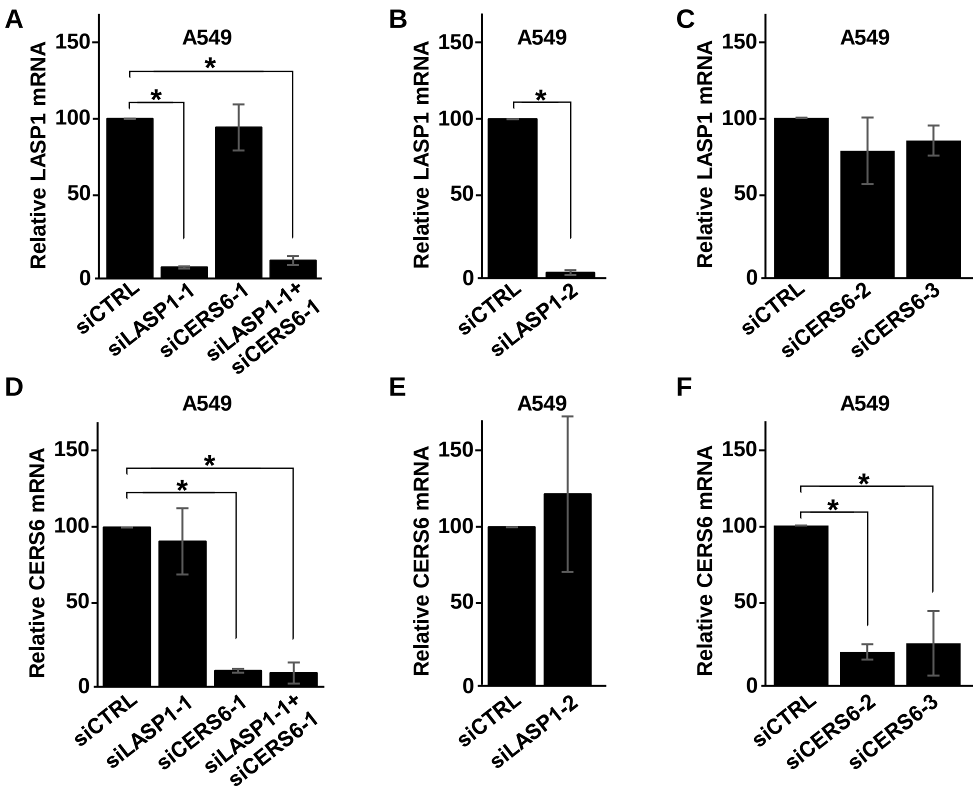

Knockdown of A549 cells was performed under the indicated conditions. LASP1 (**A-C**) and CERS6 (**D-F**) mRNA levels were determined using quantitative RT-PCR analysis, and plotted values are shown relative to siCTRL. *p<0.05, at least three independent experiments.

Figure S9 **Co-localization of LASP1 and CERS6 on actin stress fiber.**

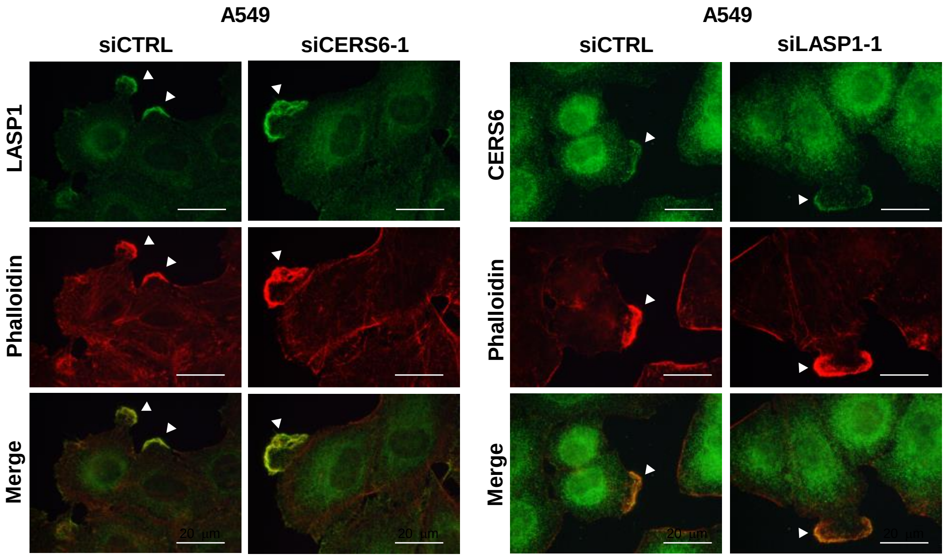

Immunofluorescent staining of phalloidin and LASP1 or CERS6 on lamellipodia of A549 cells treated with siCTRL, siCERS6-1, or siLASP1-1. Scale bar = 20 μm. Arrowhead: lamellipodia.
