# Supplementary figures and images for: LASP1, CERS6, and Actin Form a Ternary Complex That Promotes Cancer Cell Migration
Source: Cancers (Basel). 2023 May 16;15(10):2781. doi: 10.3390/cancers15102781 (PMC10216351; doi:10.3390/cancers15102781)

Figure 1

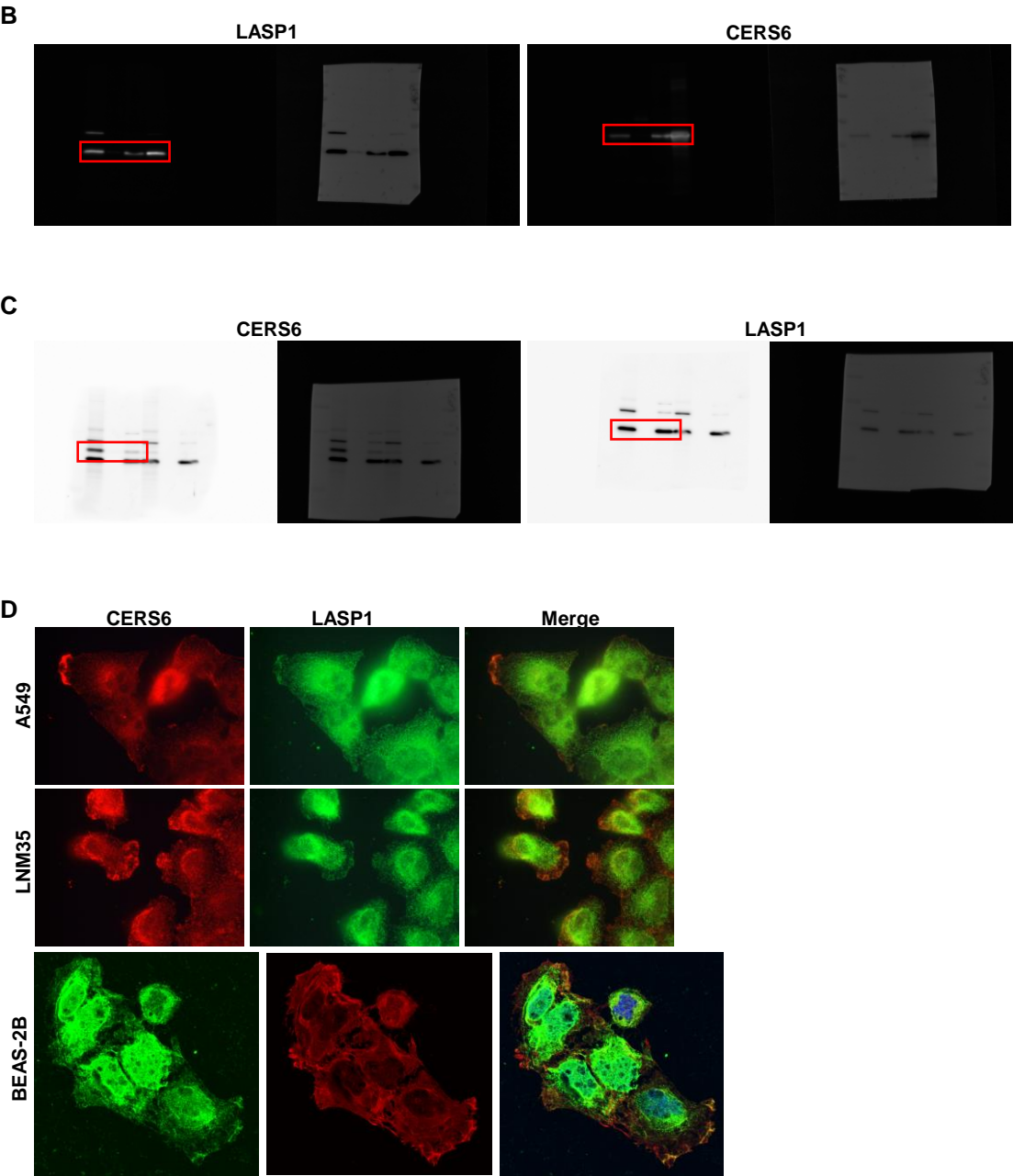

Figure 2

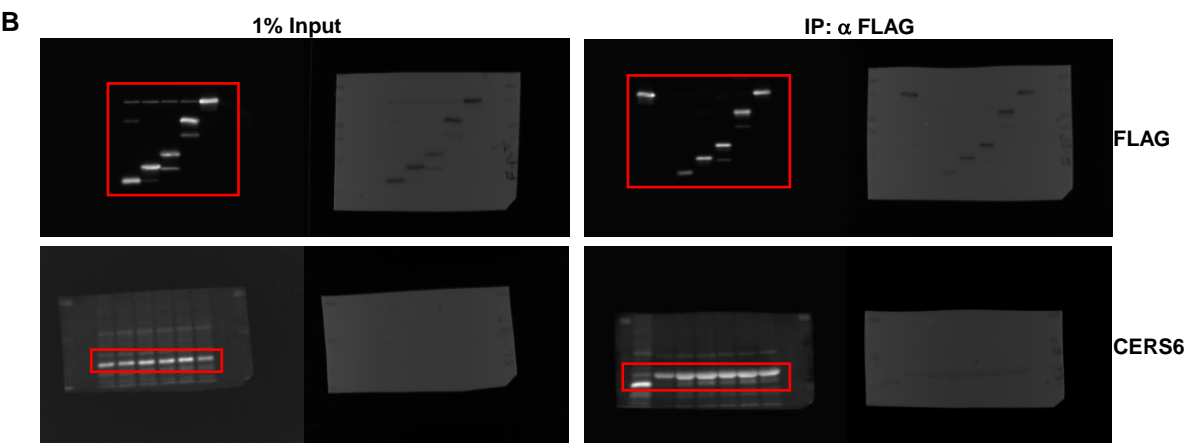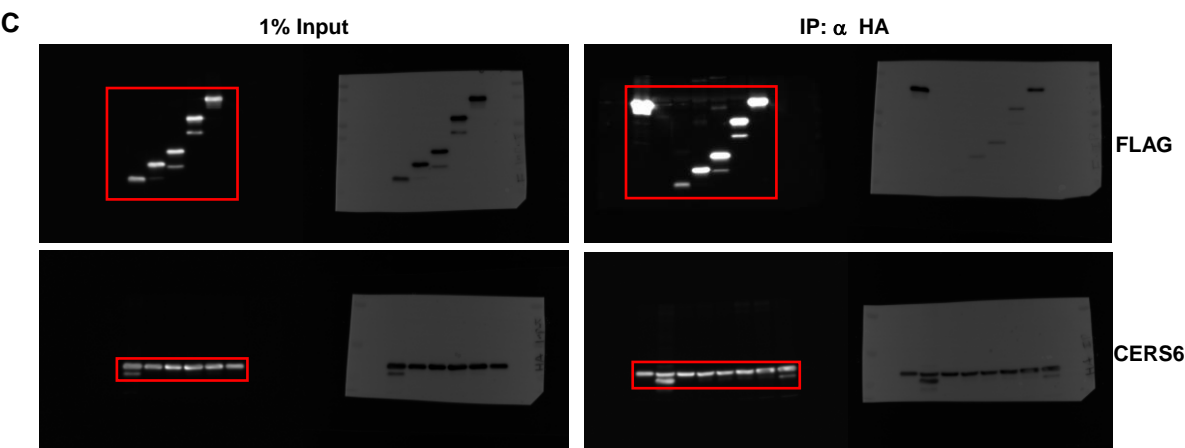

Figure 3

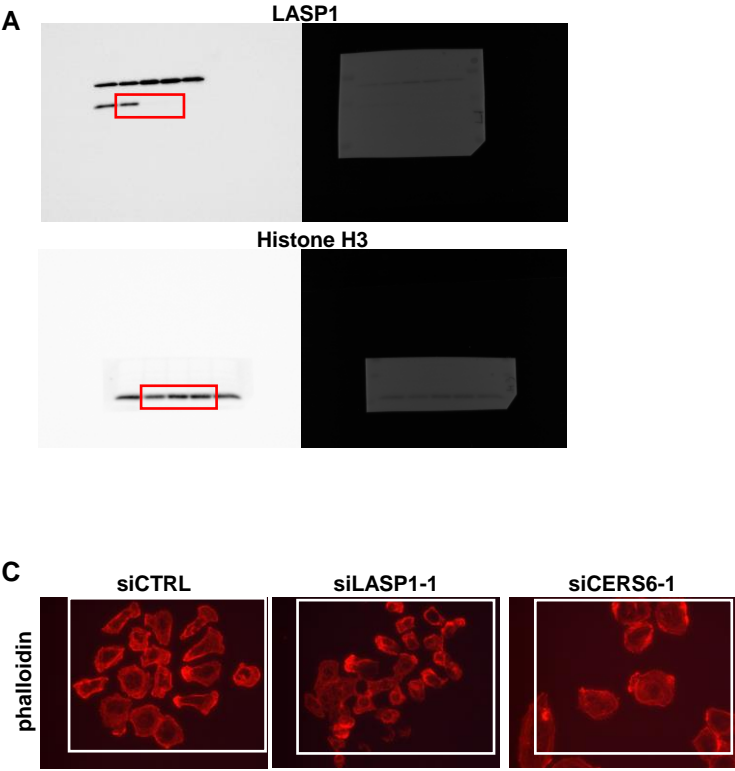

Figure 4

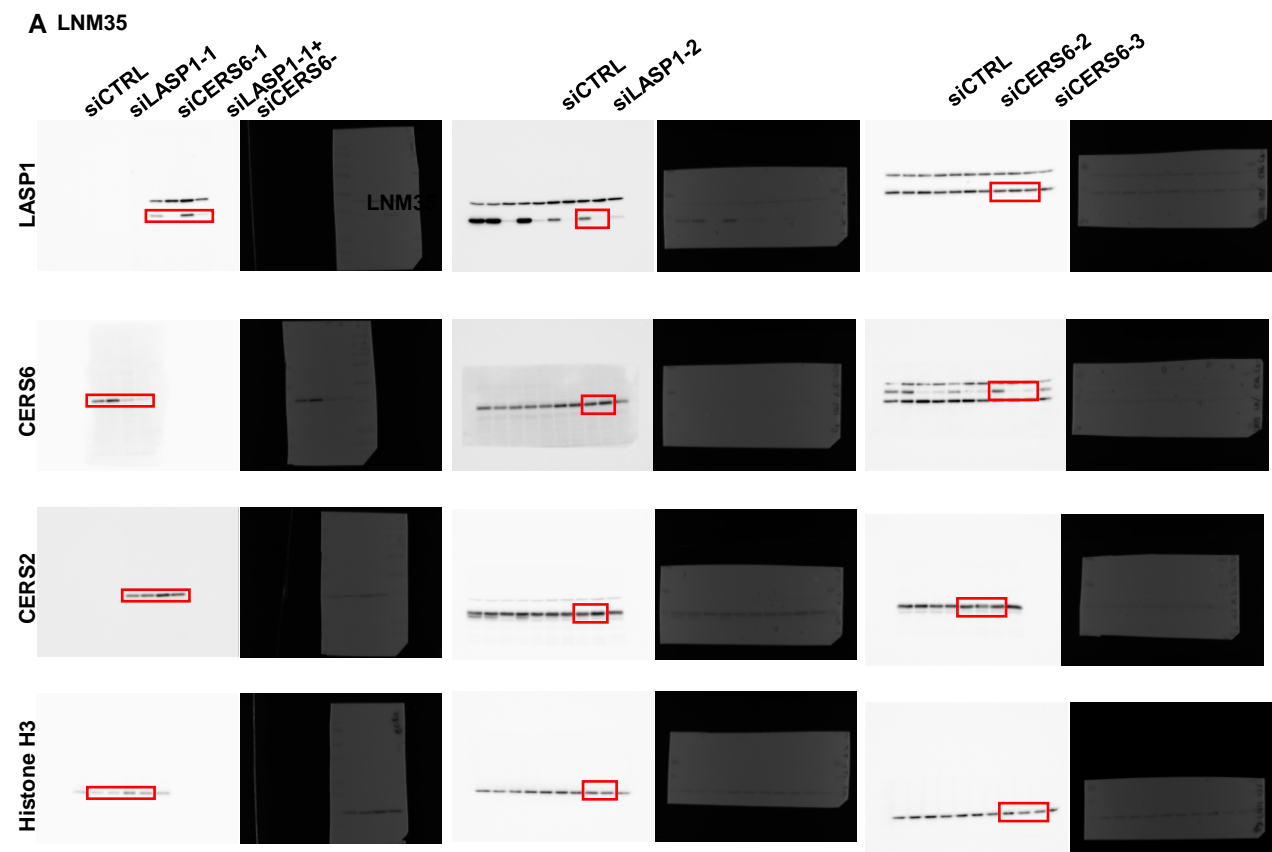

Figure 5

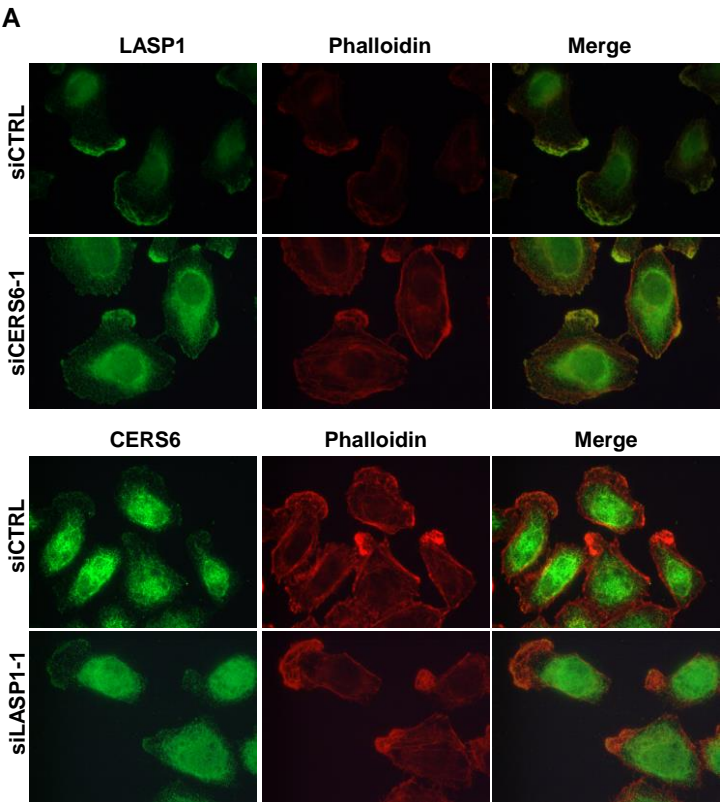

Figure 5

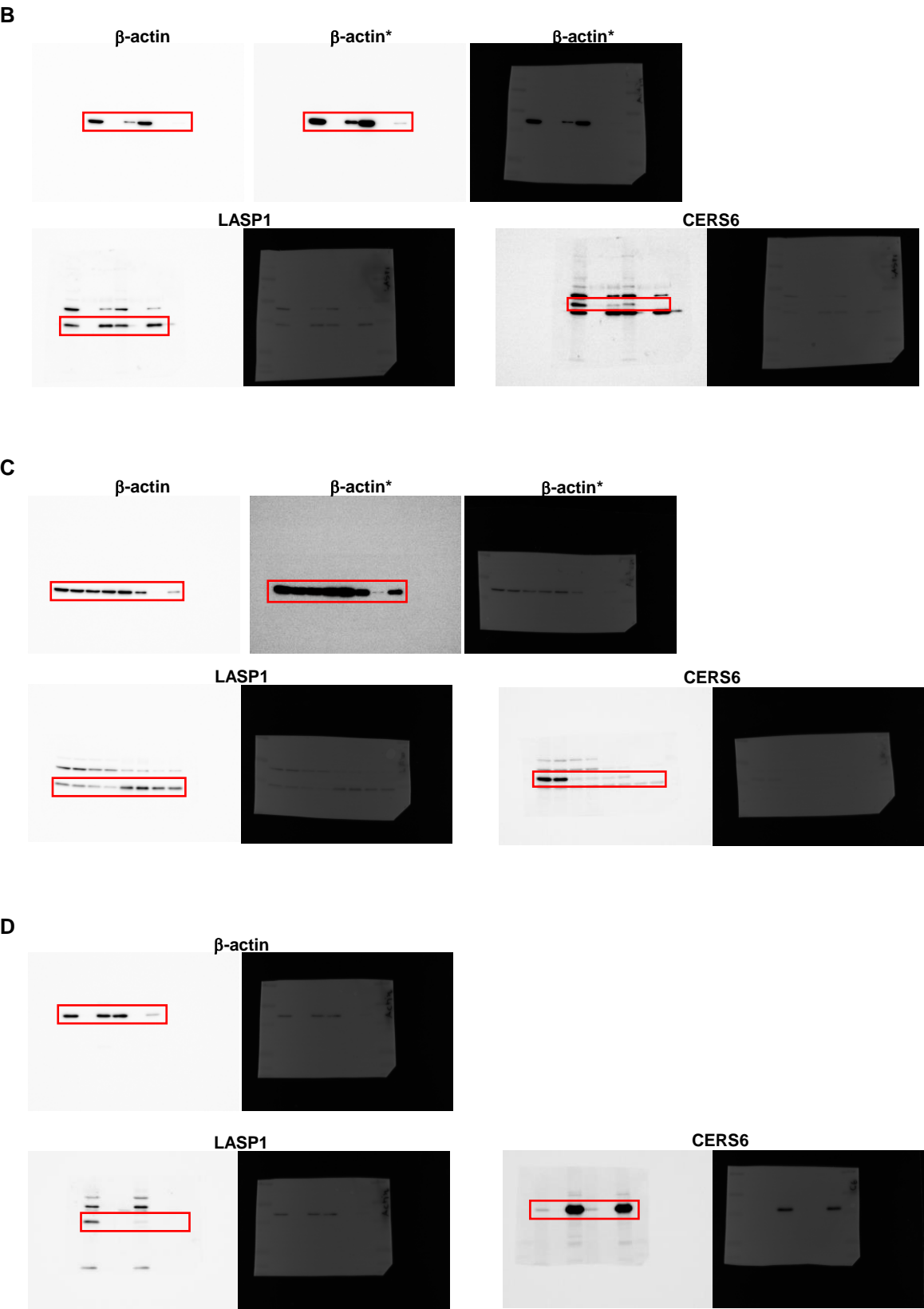

Figure S1

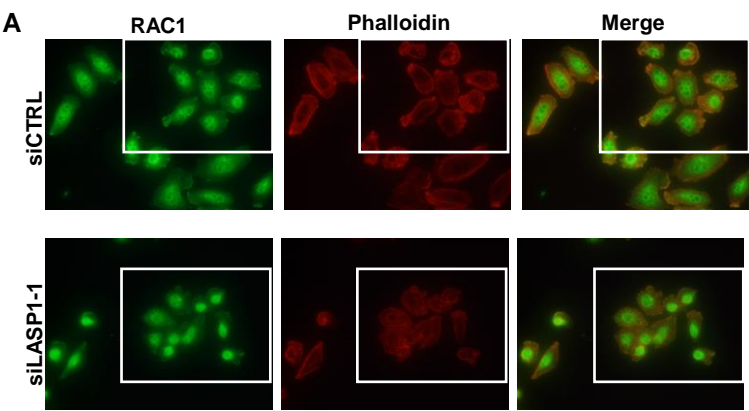

Figure S2

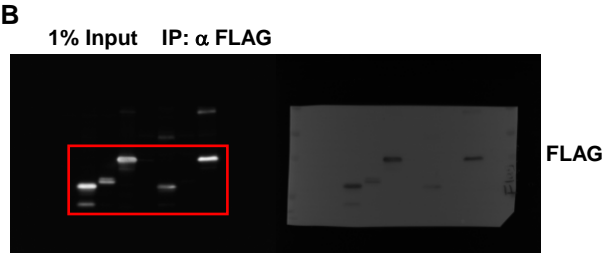

Figure S3

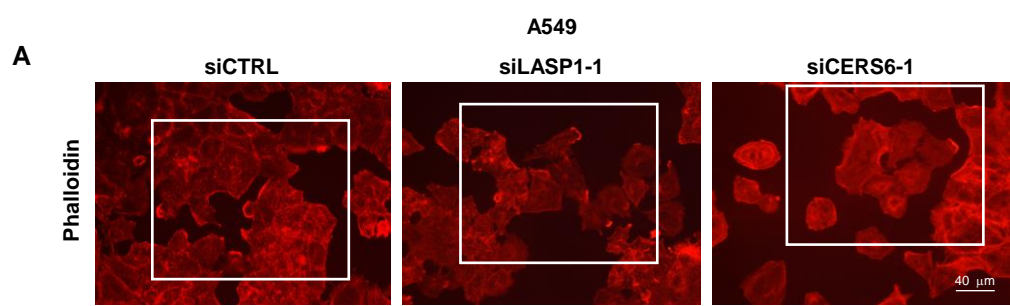

Figure S9

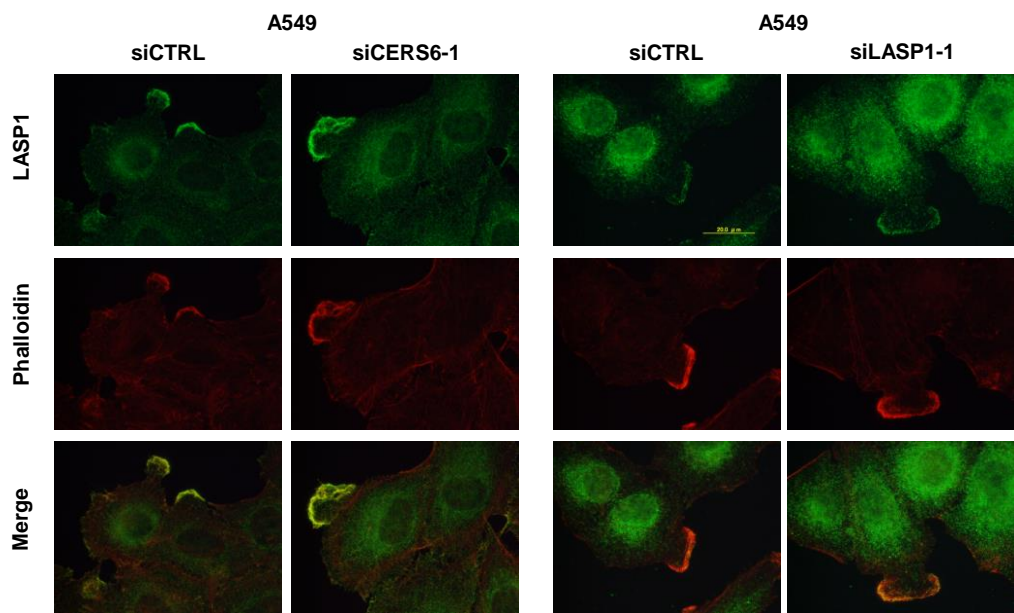

Supplement: Supplementary file 1 [file cancers-15-02781-s001.zip › Supplementary File S1.pdf]
